# Dexamethasone/β-cyclodextrin inclusion complex hydrogel for vital pulp therapy

**DOI:** 10.1007/s10266-025-01146-w

**Published:** 2025-07-09

**Authors:** Saidah Tootla, Isaac J. de Souza Araújo, Arwa Daghrery, Marco C. Bottino

**Affiliations:** 1 Department of Cariology, Restorative Sciences and Endodontics, University of Michigan School of Dentistry, 1011 N University Ave (Room 2303), Ann Arbor, MI 48109 USA; 2 Department of Pediatric and Restorative Dentistry, University of the Witwatersrand, 7 York Road Parktown, Johannesburg, 2193 South Africa; 3 College of Dentistry, University of Saskatchewan, 107 Wiggins Road, Saskatoon, SK S7N 5E5 Canada; 4 Department of Restorative Dental Sciences, School of Dentistry, Jazan University, 45142 Jazan, Saudi Arabia; 5 Department of Biomedical Engineering, College of Engineering, University of Michigan, Ann Arbor, MI USA

**Keywords:** Pulp capping, Dentin regeneration, Drug delivery, Hydrogels

## Abstract

Targeting inflammation and mineralization responses in a controlled manner for vital pulp therapies is a challenging task. Here, we prepared dexamethasone-β-cyclodextrin (DEX/ICD) inclusion complex-loaded hydrogels for pulp–dentin complex regeneration. The formation of the DEX/ICD inclusion complex was verified using Fourier-transformed infrared spectroscopy (FTIR) and X-ray diffraction (XRD). Pristine dexamethasone (DEX) and DEX/ICD were added to methacrylated gelatin (GelMA) hydrogels at a concentration of 1%. Pristine GelMA serves as the control. Drug-loaded hydrogels were evaluated for mechanical properties (*n* = 10/group), swelling and enzymatic degradation (*n* = 5/group), and drug release (*n* = 3/group). Cell proliferation, cell morphology, and mineralization assays were conducted using SHEDs. Cell proliferation was assessed on days 1, 3, and 7 using the MTS assay (*n* = 6/group). Cell morphology was analyzed via fluorescence microscopy using phalloidin and DAPI. A mineralization assay was performed using Alizarin Red at 14 and 21 days (*n* = 4/group). One-way ANOVA and Tukey’s (*α* = 0.05) post-hoc tests were applied for statistical analysis. FTIR and XRD data confirmed the formation of the DEX/ICD complex. No statistical differences were found in mechanical properties, swelling, and degradation after incorporating DEX or DEX/ICD. DEX/ICD-loaded hydrogels consistently released dexamethasone, peaking at day 14, and maintained stability until day 21. DEX-loaded GelMA release decreased after day 7. DEX/ICD-loaded GelMA exhibited higher cell proliferation than DEX-loaded GelMA on day 7 (*p* < 0.05), with no statistical differences observed for days 1 and 3 (p > 0.05). The hydrogels did not compromise cell morphology. DEX/ICD-loaded GelMA significantly enhanced mineralization potential (*p* < 0.05). The β-CD inclusion complex optimized dexamethasone delivery, and DEX/ICD-containing GelMA enhances the proliferation and mineralization capacity of dental stem cells. DEX/ICD-loaded GelMA is a promising injectable material for halting inflammation and could potentially induce mineralized tissue formation in pulp capping strategies.

## Introduction

Dental pulp exposures are commonly associated with caries progression and accidents that occur during caries removal in deep cavities. In both scenarios, it is paramount to preserve tooth vitality and stimulate tissue recovery [1]. In this way, the application of bioactive capping materials on exposed areas induces the formation of a mineralized barrier that seals the injury and prevents an irreversible condition [2–4]. Calcium hydroxide and calcium silicate-based cements are gold-standard materials for pulp capping due to their antibacterial activity, partial solubilization of the dentin matrix that releases growth factors, and their stimulation of progenitor cell differentiation for reparative dentin secretion [5–8]. However, dentin formed after pulp capping is highly soluble and non-homogeneous, with osteodentin characteristics, which compromises the long-term stability of the tissue [1, 5, 7, 9]. Moreover, pulp capping materials have drawbacks related to the formation of a superficial necrotic zone on the pulp due to their caustic pH [4, 10]. These limitations underscore the need for effective tissue engineering strategies to regulate the inflammatory response and repair the dentin–pulp complex.

In that regard, dexamethasone (DEX) is a synthetic glucocorticoid with anti-inflammatory activity that plays an adjuvant role in guiding stem cells toward osteogenic and odontogenic differentiation [11]. Dexamethasone participates in different stages of mesenchymal stem cells’ osteogenic differentiation by regulating the expression of specific bone markers [12–14]. Unfortunately, high concentrations of dexamethasone have cytotoxic effects on particular cell lineages [15, 16], which limits its application in regenerative strategies. Furthermore, dexamethasone is lipophilic and hard to dissolve in aqueous drug delivery vehicles such as hydrogels. Therefore, it is necessary to find alternatives for controlled drug release in therapeutic approaches.

β-Cyclodextrin is an alternative to improve the solubility of lipophilic drugs [17] due to its amphiphilic cone-shaped structure, where the inner portion is hydrophobic, and the external surface is hydrophilic [17]. β-Cyclodextrin can entrap lipophilic drugs by forming inclusion complexes through non-covalent bonds [18]. The formation of β-cyclodextrin inclusion complexes with corticosteroids and other lipophilic drugs has been investigated in previous studies to enhance drug solubility and improve therapeutic outcomes in various fields [19–22].

Optimizing drug solubility enables adequate conditions for dissolution into drug carriers, supporting regenerative strategies. Methacrylated gelatin hydrogel (GelMA) has been applied as a scaffold in tissue regeneration of soft and mineralized tissues in the craniofacial complex [23–26]. GelMA functions as a permeable matrix that mimics native tissue conditions for stem cell differentiation [27]. Herein, we prepared dexamethasone/β-cyclodextrin inclusion complexes to enhance the solubility of dexamethasone in GelMA hydrogels, thereby facilitating the sustained release of dexamethasone as a direct pulp capping strategy.

## Materials and methods

### Materials

Dexamethasone (> 99%, Tokyo Chemical Industry Co., Ltd., Tokyo, Japan). β-Cyclodextrin, type A gelatin from porcine, methacrylic anhydride (MA), and Alizarin Red (Sigma-Aldrich, St. Louis, MO, USA). Lithium phenyl-2,4,6-trimethylbenzoylphosphinate (LAP L0290, TCI America Inc., Portland, OR, USA). Alpha-modified Eagle's Medium (α-MEM), fetal bovine serum (FBS), and penicillin–streptomycin (Gibco-Invitrogen, San Diego, CA, USA). TRITC-conjugated phalloidin and DAPI (Millipore, Burlington, MA, USA). CellTiter 96 AQueous One Solution Reagent (Promega Corporation, Madison, WI, USA).

### Inclusion complex preparation

The DEX/β-CD inclusion complex (DEX/ICD) was prepared through freeze-drying as previously described [22]. Briefly, β-CD was dissolved in distilled water, and DEX was added to the solution at a 1:1 molar ratio. The mixture was then stirred for 24 h. The solution was kept at – 80 °C for an additional 48 h, and then freeze-dried for 72 h. The phase composition of DEX/ICD was characterized using X-ray Diffraction (XRD, Rigaku Ultima IV diffractometer, Rigaku Americas Co., The Woodlands, TX, USA) with Cu Kα (*λ* = 1.54 angstroms) scanned in 2 theta (2*θ*) range from 5° to 45°. The chemical structure was further confirmed using Fourier-Transformed Infrared Spectroscopy (FTIR Spectrometer, Nicolet iS50 FTIR, ThermoFisher Scientific Inc., Waltham, MA, USA) with 32 scans between 4000 and 600 cm^−1^ regions and a 4 cm^−1^ resolution.

### GelMA synthesis and drug incorporation

Type A gelatin was dissolved in DPBS at 50 °C while stirring for 1 h (180 rpm). Then, 8 mL of MA was added dropwise to the solution and stirred for 2 h. The solution was subsequently dialyzed against deionized (DI) water at 40 °C using dialysis membranes with a molecular weight cutoff of 12–14 kDa. The dialyzed GelMA was filtered and freeze-dried. To incorporate the drugs, 10% GelMA (*w*/*v*) was dissolved in DPBS, and three groups were prepared: GelMA (Pristine); G-DEX (GelMA solution containing pristine DEX at 1% (*w*/*v*)); and G-DEX/ICD (GelMA solution containing DEX/ICD dissolved at 1% weight of DEX). Subsequently, LAP at 0.5% was added, and the vials were stirred overnight. The mixtures were transferred to custom molds and light-cured using Bluephase Style (Ivoclar-Vivadent, Amherst, NY, USA) for 30 s. The cured samples were removed from the molds and used for further characterization.

### Mechanical characterization

To evaluate the mechanical properties, cylindrical-shaped (10 × 3 mm) samples of the hydrogels (*n* = 10/group) were incubated in DPBS at 37 °C for 24 h before testing. Blot-dried samples were subjected to unconfined compression at a crosshead speed of 1 mm/min (Expert 5601, ADMET Inc., Norwood, MA, USA). The stress–strain curves and the compressive modulus were recorded.

### Swelling and enzymatic degradation

Swelling ratios were determined by incubating hydrogel samples (6 × 2 mm) in DPBS for 24 h at 37 °C. Then, the samples (*n* = 3/group) were blot-dried and weighed to obtain their wet weights (Ww). The samples were freeze-dried, and the dry weights (Wd) were registered. The swelling ratio was calculated using the equation: Sw = (*W*_*w*_ – *W*_*d*_)/*W*_*d*_. For enzymatic degradation, after recording the initial weights (*W*_0_), the samples (*n* = 5/group) were placed in 2 mL of type I collagenase-containing solution (1 U/mL) at 37 °C. The solution was changed every 3 days to maintain enzymatic activity. At predetermined intervals, samples were removed from the solution, blot-dried, and weighed (*W*_t_). The degradation ratio was calculated using the following equation: Degradation (%) = (*W*_t_/*W*_0_) × 100.

### Drug release

To determine the drug’s release kinetics, cylinder-shaped samples (6 × 2 mm) of G-DEX and G-DEX/ICD (n = 3/group) were each immersed in 5 ml of PBS for 21 days. On days 1, 3, 7, 14, and 21, 500 µl of aliquots was taken, and fresh PBS was added to replace the extracted volume. The absorbance of dexamethasone from the collected aliquots was measured using a microplate reader (SpectraMax iD3, Molecular Devices LLC, San Jose, CA, USA) at 240 nm. Concentration was determined by comparing the absorbance values to a standard curve.

### Cell culture

Stem cells from human exfoliated deciduous teeth (SHEDs) were used in biological assays. SHEDs were cultured in α-MEM, supplemented with 10% FBS and 1% penicillin/streptomycin. The cells were incubated in a 5% CO_2_ atmosphere at 37 °C. For all assays involving cells, 100 µL of GelMA, G-DEX, and G-DEX/ICD was pipetted into the bottom of well plates and light-cured. The cells were seeded at specific densities as detailed in the experiments.

### Cell viability and morphology

SHEDs were seeded at 1 × 10^4^ cells/well in 96-well plates (*n* = 6/group) on top of the hydrogels. After each time point (1, 3, and 7 days), the wells were washed with PBS, and then MTS solution was incubated for 2 h at 37 °C and 5% CO_2_. The absorbance of the soluble formazan was read at 490 nm using a microplate reader (SpectraMax iD3).

Cell morphology was assessed through fluorescence microscopy. SHEDs were cultured on top of the hydrogels (2 × 10^4^ cells/well) in a 48-well plate (n = 3/group) for 1 and 3 days. Then, the cells were fixed in 4% paraformaldehyde for 30 min, washed in PBS, and permeabilized using 0.1% Triton X-100 for 5 min. Samples were blocked using 1.5% bovine serum albumin in PBS for 30 min and stained with DAPI for the nucleus and Phalloidin for the cytoplasm. After staining, the wells were imaged using a fluorescence microscope (Carl Zeiss Meditec AG, Jena, Germany) at 20 ×.

### Mineralization assay

Mineralization potential was determined using Alizarin Red staining (ARS) after culturing 3 × 10^4^ cells/well on top of the hydrogels in a 24-well plate in osteogenic medium for 14 and 21 days. Cells were fixed in 4% paraformaldehyde for 1 h, washed twice with PBS, and stained for 20 min with a 40 mM ARS solution. Relative quantification was performed by dissolving the mineralization spots in 500 µL of CPC and measuring the absorbance at 562 nm using a microplate reader (SpectraMax iD3).

### Statistical analysis

Statistical analyses were performed in GraphPad Prism (GraphPad Software, San Diego, CA, USA) using one-way ANOVA and Tukey’s post-hoc (α = 0.05).

## Results

**Fig. 1:**
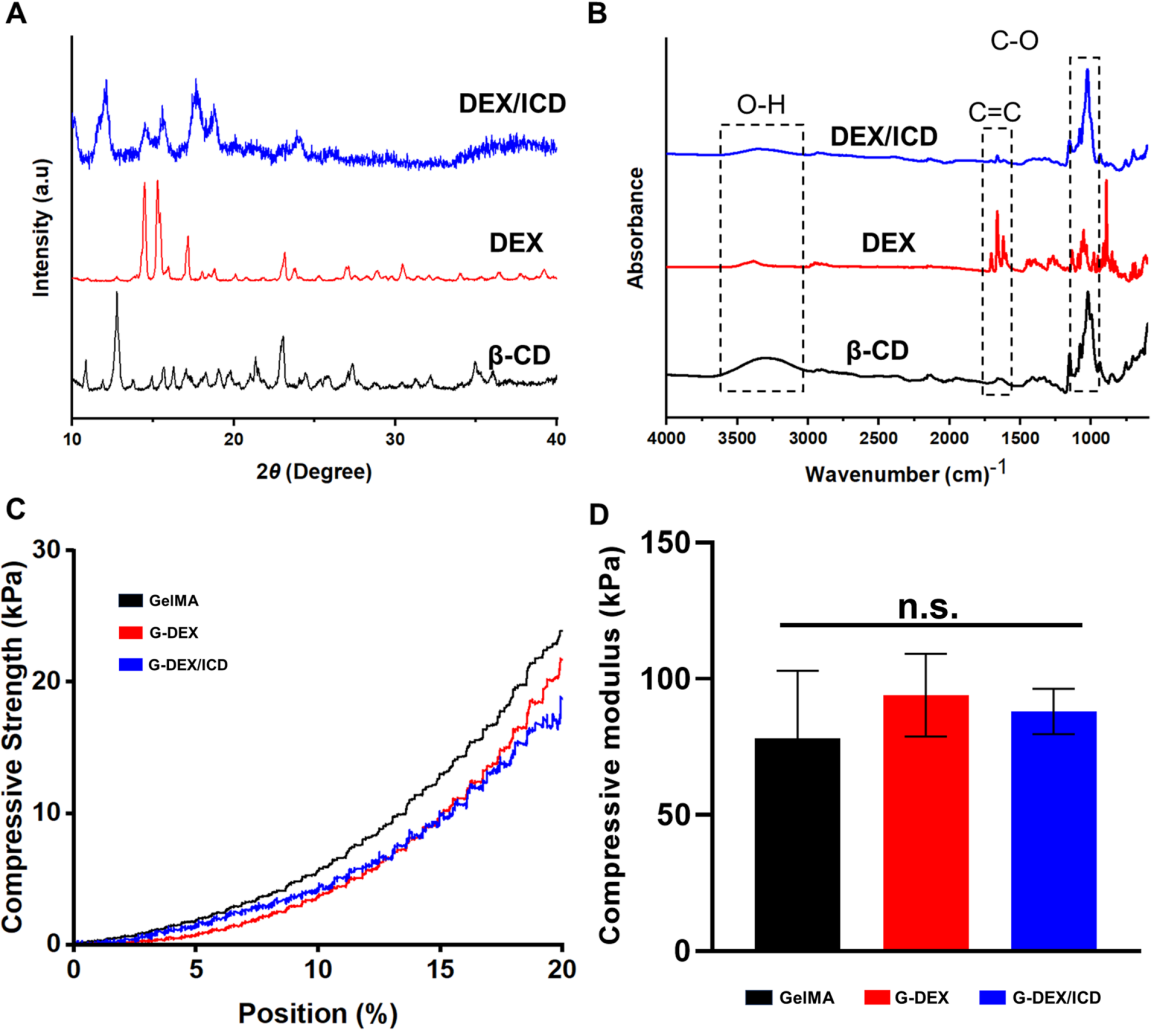
**A** XRD patterns at 2θ degree of pristine DEX, β-CD, and DEX/ICD formed after lyophilization. The crystalline peak of dexamethasone is evidenced in the 15° region. A more amorphous and noisier pattern is evidenced for DEX/ICD, where the 15° peak is considerably broadened, while β-CD presents a peak at 12° that is also broadened in DEX/ICD. **B** FTIR spectra of the pristine compounds and DEX/ICD. The dashed areas indicate the C=C stretching peaks at the 1620–1640 cm^−1^ characteristic of dexamethasone are also present for DEX/ICD. The C–O stretching at 1000–1100 cm^−1^ region corresponding to β-CD is maintained for DEX/ICD, while the peak for hydroxyl (OH) stretching is broadened (3000–3400 cm^−1^). **C** Compressive strength curves of GelMA, G-DEX, and G-DEX/ICD. Incorporating DEX or DEX/ICD caused a slight reduction in compressive strength. **D** Mean and standard deviation of the compressive modulus of GelMA, G-DEX, and G-DEX/ICD, where a slight increase was evidenced with no significant differences among the groups (*p* > 0.05). n.s. (No statistical significance)

Figure 1A, B presents the XRD patterns and FTIR spectra of pristine dexamethasone (DEX), β-cyclodextrin (β-CD), and DEX/β-cyclodextrin inclusion complexes (DEX/ICD). The conventional crystalline peak of dexamethasone is evident in the 15° region at 2θ. A more amorphous and noisier pattern is observed for DEX/ICD, where the 15° peak is significantly broadened. The β-CD pattern shows a peak in the 12° region, which is also broadened in DEX/ICD **(**Fig. 1A**)**. The FTIR spectra reveal the presence of C–C stretching peaks at 1620–1640 cm^−1^, characteristic of dexamethasone, and this feature is also present in DEX/ICD. Meanwhile, the C–O stretching in the 1000–1100 cm^−1^ region of the spectra corresponding to β-CD is maintained at a similar intensity for DEX/ICD, but the peak of the hydroxyl stretching is broadened (3000–3400 cm^−1^) **(**Fig. 1B**)**. The compressive strength curves and the compressive modulus of GelMA, G-DEX, and G-DEX/ICD are illustrated in Fig. 1C, D. The incorporation of the drug, either in its free form or after the formation of the inclusion complexes, resulted in a slight reduction in compressive strength (Fig. 1C). Simultaneously, a slight increase was observed for the compressive modulus, though there were no significant differences among the groups (Fig. 1D).

**Fig. 2:**
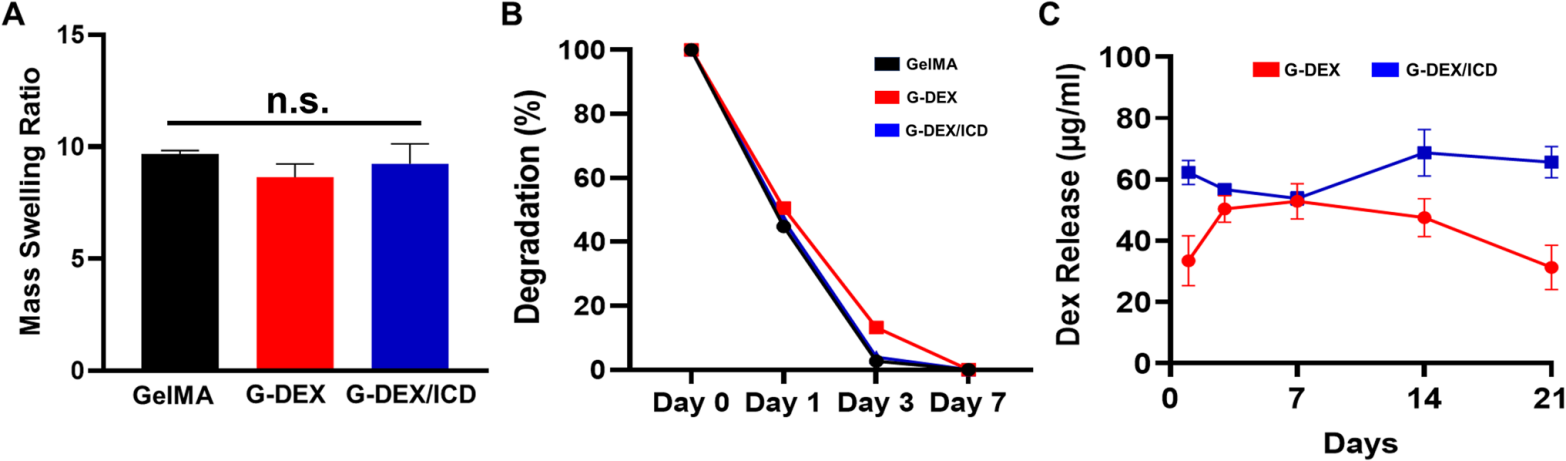
**A** Mean and standard deviation of the swelling ratios for GelMA, G-DEX, and G-DEX/ICD. n.s. (No statistical significance). **B** Enzymatic degradation profiles of GelMA, G-DEX, and G-DEX/ICD up to 7 days. **C** Mean and standard deviation of the release profiles of G-DEX and G-DEX/ICD up to 21 days. G-DEX/ICD presented a higher release of dexamethasone compared to G-DEX throughout all the time points. The peak of release for G-DEX was on day 7 with a continuous decrease for the subsequent time points. G-DEX/ICD presented a slight drop in release from day 1 to day 7. The peak of release was evidenced on day 14 and sustained until 21 days

Moreover, no impact on the swelling ratio and enzymatic degradation was observed for G-DEX and G-DEX/ICD (Fig. 2A, B). Regarding the release profile illustrated in Fig. 2C, G-DEX/ICD exhibited a sustained higher dexamethasone release than G-DEX at all time points. The peak release for G-DEX occurred on day 7, followed by a continuous decrease at subsequent time points. Meanwhile, G-DEX/ICD presented a slight drop in release from day 1 to day 7. The peak of release was evidenced on day 14 and sustained until day 21.

**Fig. 3:**
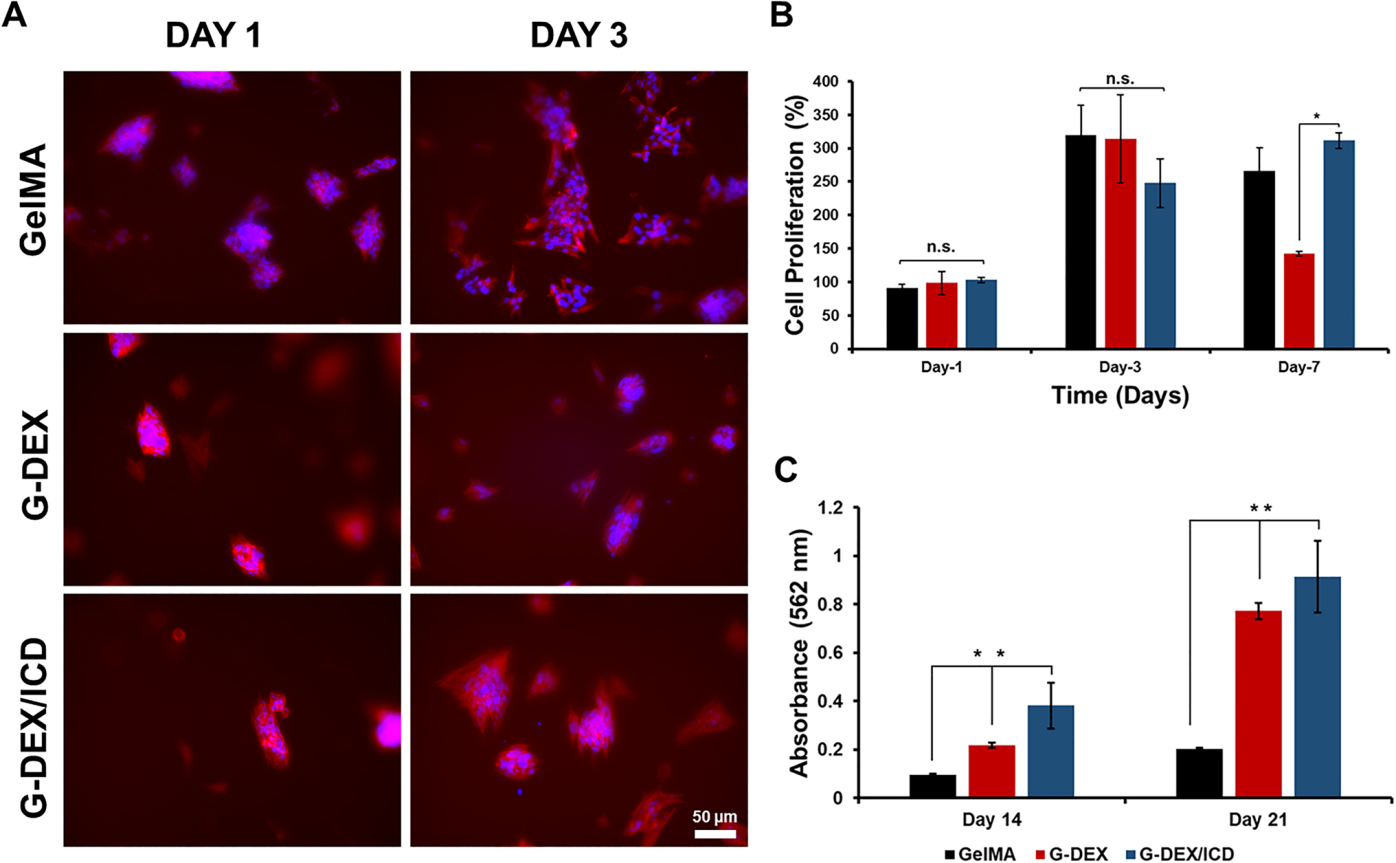
**A)** Fluorescence microscope images for cell morphology using DAPI and Phalloidin staining. **B** Cell proliferation percentage at different time points. **C** Alizarin Red quantification for osteogenic differentiation at 14 and 21 days. Asterisks (*) indicate statistical differences between groups. ns (no statistical significance)

Representative images of cell morphology are shown in Fig. 3A. On the first day of culture, agglomerates of cells can be seen attaching to the surface of the gels with discrete spreading. By day 3, the characteristic filopodia extensions of SHEDs are visible in all groups, accompanied by an apparent increase in cell number, particularly notable in the pristine GelMA group. Figure 3B presents the mean values and standard deviation of the cell proliferation assay. There was no statistical significance in cell proliferation at 1 and 3 days. However, a significant reduction was observed for G-DEX compared to G-DEX/ICD on day 7. The quantitative measurements of mineralization for days 14 and 21 are displayed in Fig. 3C. The incorporation of dexamethasone significantly enhanced osteogenic differentiation compared to pristine GelMA, with osteogenic activity increasing from day 14 to 21 for both G-DEX and G-DEX/ICD. Notably, G-DEX/ICD exhibited the highest mineralization potential at both time points (*p* < 0.05).

## Discussion

Pinpoint pulp exposures resulting from caries progression or mechanical removal of affected tissue necessitate the use of direct pulp capping or other vital pulp therapy strategies to preserve tooth vitality [1]. Clinical conditions, such as the size, number, and location of the exposure, bleeding control, and the degree of pulp inflammation, are determinants in decision-making. Despite the relatively high success rates, the current evidence is inconclusive regarding which clinical approach (direct pulp capping, partial pulpotomy, or full pulpotomy) is more effective for each scenario [28, 29].

Regarding material selection, hydraulic calcium- and silicate-based cements tend to replace calcium hydroxide as the preferred materials for sealing pulp exposures in vital pulp therapy after hemorrhage control [30]. However, the drawbacks of high pH and the initial inflammatory response caused by these materials persist, leaving room for enhancements through more cytocompatible bioinductive materials. Here, we propose an innovative approach to potentially manage inflammation and promote pulp–dentin complex regeneration through the local delivery of dexamethasone, optimizing the solubility and release profile of the drug by forming DEX/ICD for incorporation into GelMA hydrogels.

The formation of the inclusion complex was confirmed using XRD and FTIR data, where typical peaks of DEX and β-CD are evident for DEX/ICD, despite slight changes in intensity, which support the guest–host interaction. These variations in FTIR and XRD peaks are possibly related to chemical changes in the structure of cyclodextrin aggregates after conjugation, as well as the reduced ability of dexamethasone molecules to maintain crystalline organization after being trapped in β-CD’s cone-shaped structure [22, 31, 32]. In addition, the incorporation of pristine DEX and DEX/ICD into GelMA hydrogels did not significantly impact the compressive strength or affect the compressive modulus. The same behavior was observed for the swelling ratio and degradation profiles. Thus, GelMA is a suitable carrier for DEX and DEX/ICD delivery. Nonetheless, for critical scenarios of pulp inflammation after caries removal, one might consider delivery mechanisms that take longer to degrade to extend the effects of dexamethasone, such as utilizing GelMA's versatility by altering the stiffness of the gel or synthesizing electrospun membranes with GelMA as the base polymer [33] for efficient incorporation of DEX/ICD [22]. The tunable characteristics of GelMA, its low toxicity, and the potential use of photo-initiators activated by dental light-curing units further support its application in dental pulp regeneration strategies [23, 24].

Notably, the release profile was considerably altered when comparing DEX and DEX/ICD over 21 days. Dexamethasone released from G-DEX/ICD was higher than that from G-DEX, peaking on day 14 and maintaining sustainability. In contrast, G-DEX exhibited an initial increase in dexamethasone release, which significantly declined after day 7. These findings reinforce that β-CD conjugates enhance the delivery of lipophilic drugs [34] and improve their stability in aqueous solutions [35]. The lack of a guest–host structure in G-DEX causes dexamethasone agglomerates to form in the bulk of GelMA hydrogels due to the corticosteroid's lipophilic nature, which contributes to the unstable profile and substantial drop after the peak release.

Furthermore, no significant impact was observed on cell proliferation and morphology until day 3. Typical filopodia extensions of SHEDs were noted for all groups, with a clear increase in cell number from day 1 to day 3. However, there was a substantial decrease in cell proliferation for G-DEX on day 7, possibly related to the peak release of dexamethasone, which promotes unfavorable conditions for SHEDs to maintain their proliferative state. Such a critical environment caused by high concentrations of dexamethasone aligns with reports regarding the adverse effects of DEX on some cells [15, 16]. Meanwhile, although the drug released from G-DEX/ICD is higher than that from G-DEX at 7 days, no reduction in cell viability and proliferation was observed. Therefore, one could suggest that either the dissociation process from β-CD or the conjugation itself mitigates the adverse effects of high concentrations of dexamethasone on cells.

The sustained release of DEX over 3 weeks presents clinical potential as a delivery system for resident dental pulp stem cell differentiation [22], as the enhanced solubility and gradual release support odontogenic and osteogenic differentiation in a dose-dependent manner [15]. Notably, dexamethasone released from G-DEX/ICD significantly increased the osteogenic differentiation of SHEDs, showing the highest mineralization potential on days 14 and 21. However, incorporating DEX/ICD at concentrations above 5% into nanofibers reduces osteogenic differentiation and ALP activity [22]. Therefore, maintaining a safe range with soluble vehicles like hydrogels enables osteogenic differentiation at lower concentrations and could mitigate the risk of toxicity in injured dental pulps after exposure. In addition, multiple factors influence decision-making for vital pulp therapies in clinical settings [30], and further investigation is needed to evaluate the performance of our hydrogel containing DEX/ICD in inflammatory scenarios after caries removal and to compare it with gold-standard pulp capping materials.

It is also important to highlight that vital pulp therapy is often necessary due to deep caries reaching the pulp, and decontamination of the exposed tissue is crucial for the success of the treatment, which was not tested in our model. Disinfection protocols in regenerative endodontics utilize sodium hypochlorite, chlorhexidine, or antibiotic pastes [36], but concerns and discussions about the balance between the antimicrobial activity and toxicity of these agents always arise. In this regard, antibacterial strategies employing localized delivery of cytocompatible drugs, such as natural compounds [37], could be combined with our anti-inflammatory therapeutics in a dual-delivery system for antibacterial effects and enhanced tissue response to minimize the use of toxic materials in regenerative endodontics. In addition, for larger defects like full pulpotomies, one may consider the need for revascularization and the formation of a new coronal pulp. Such strategies could involve lyophilized scaffolds that may benefit from contact with blood and stem cells, which has been verified elsewhere for regenerative endodontics [38]. However, some concerns may remain regarding the degradability of these constructs in a hierarchical manner for optimized regeneration. Therefore, using bioprinted scaffolds is also an alternative approach [23, 39], as technology in the field continues to advance towards biofabrication [40]. However, this was not the focus of our investigation, and other studies may explore these possibilities in the future.

Although this model was tested purely in vitro, the formation of DEX/ICD optimized dexamethasone solubility and release from GelMA. Since DEX/ICD-loaded GelMA significantly stimulated SHEDs’ proliferation and osteogenic differentiation, future orthotopic in vivo studies are necessary to elucidate the local efficacy of G-DEX/ICD in pulp exposures. More specifically, validation in vivo models may include traditional rodent models of pulp exposure [33] or more complex studies in large animals with previously induced pulp inflammation [41] to compare our DEX/ICD-containing hydrogels with the current gold-standard capping materials. These comparisons are also necessary due to potential changes in the inflamed pulp and the well-known clearance ability of the tissue that enables conventional calcium hydroxide and calcium- and silicate-based cements to form dentin bridges, as pulp conditions are a determining factor in decision-making and prognosis of vital pulp therapies [42].

## Conclusion

The formation of dexamethasone/β-CD inclusion complexes is suitable for improving drug solubility and controlling the release profile from GelMA hydrogels. The sustained release of dexamethasone from DEX/ICD-loaded GelMA enhances the proliferation and mineralization of dental stem cells, making this delivery system a promising method for pulp capping strategies.

## Data Availability

The data that support the findings of this study are available from the corresponding authors upon reasonable request.
